# Nobiletin inhibits cell growth through restraining aerobic glycolysis via PKA‐CREB pathway in oral squamous cell carcinoma

**DOI:** 10.1002/fsn3.1634

**Published:** 2020-05-21

**Authors:** Chong‐Xiang Lin, Cheng‐Wei Tu, Yi‐Ke Ma, Peng‐Cheng Ye, Xia Shao, Zhao‐An Yang, Yi‐Ming Fang

**Affiliations:** ^1^ Department of Stomatology The First Affiliated Hospital of Wenzhou Medical University Wenzhou China; ^2^ School and Hospital of Stomatology Wenzhou Medical University Wenzhou China

**Keywords:** aerobic glycolysis, CREB, mitochondrial, Nobiletin, PKA

## Abstract

**Background/Aim:**

Nobiletin is a polymethoxylated flavone enriched in Citrus and is used as an important drug in traditional Chinese medicine for various kinds of diseases. Among its multiple functions, it has shown that nobiletin inhibits proliferation of various cancer cells. However, it is unclear whether nobiletin inhibits the growth of oral squamous cell carcinoma (OSCC) cells.

**Materials and Methods:**

We explored the antitumor effects of nobiletin in TCA‐8113 and CAL‐27 oral squamous cells. The Cell Counting Kit‐8 (CCK8) assay was used to measure cell vitality. Flow cytometry was performed to measure the number of cells in the various phases of the cell cycle. PCR and Western blot were applied to determine mRNA and protein expression, respectively.

**Results:**

Nobiletin inhibited proliferation of TCA‐8113 and CAL‐27 cells via inducing cell cycle arrest at the G1 phase. In addition, the levels of phosphorylated‐PKA and phosphorylated‐CREB were reduced in nobiletin‐treated TCA‐8113 and CAL‐27 cells. Importantly, our results showed that nobiletin treatment resulted in impaired mitochondrial function and altered glucose consumption, and pyruvate and lactate production. Lastly, nobiletin was found to inhibit the generation of xenografts in vivo. Interestingly, administration of 50 μmol/L Sp‐cAMP, a potent PKA activator, rescued all phenotypes caused by nobiletin.

**Conclusions:**

Nobiletin inhibits OSCC cell proliferation in a mitochondria‐dependent manner, indicating that it may have a promising role in cancer treatment and attenuation of drug resistance.

## Graphical abstract

In summary, our data validated the antitumor function of nobiletin in OSCC cells through inhibiting proliferation. Additionally, our data demonstrated that nobiletin functions via PKA/CREB pathway and modulates mitochondrial function.

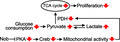

## 1 INTRODUCTION

Nobiletin (5, 6, 7, 8, 3’, 4’‐hexamethoxyflavone) is a polymethoxylated flavone that is present in dietary fruits such as oranges and has been an important drug in traditional Chinese medicine. Nobiletin has been demonstrated to exhibit anti‐inflammatory (Imada et al., 2008; Wu, Zhou, Tao, & Li, 2006), anti‐atherosclerotic (Whitman, Kurowska, Manthey, & Daugherty, 2005), and anti‐allergenic (Jang et al., 2013; Kobayashi & Tanabe, 2006) activities. Interestingly, a critical role of nobiletin in circadian rhythms regulation has been demonstrated lately (Da, Liu, Zhan, Liu, & Wang, 2016; Nohara et al., 2015; Shinozaki et al., 2017). Recently, increasing attention has been given on the anti‐cancer effects of nobiletin (Chen, Ono, Takeshima, & Nakano, 2014; Chen, Creed, et al., 2014; Ma et al., 2015; Moon & Cho, 2016; Sp et al., 2017; Sp et al., 2018; Surichan, Arroo, Ruparelia, Tsatsakis, & Androutsopoulos, 2018). It has been reported that nobiletin inhibits proliferation of multiple types of cancer cells including glioma (Aoki, Yokosuka, Mimaki, Fukunaga, & Yamakuni, 2013; Lien et al., 2016), acute myeloid leukemia (Hsiao et al., 2014), hepatocellular carcinoma (Ohnishi et al., 2004), colon cancer (Morley, Ferguson, & Koropatnick, 2007; Qiu et al., 2010; Silva et al., 2018; Wu, Song, et al., 2018; Wu et al., 2015), ovarian cancer (He, Li, Rankin, Rojanasakul, & Chen, 2015), and breast cancer cells (Chen, Creed, et al., 2014; Morley et al., 2007; Rahideh et al., 2017; Rahideh et al., 2017; Sp et al., 2017; Surichan et al., 2018). However, the role that nobiletin plays in oral squamous cell carcinoma is poorly understood.

Multiple signaling pathways have been involved in nobiletin‐mediated metabolites modulation (Huang et al., 2016). Among the various pathways, cAMP/PKA/ERK/CREB (cyclic adenosine monophosphate/protein kinase A/extracellular‐signal‐regulated kinase/cAMP response element‐binding protein) signaling plays essential roles in nobiletin‐regulated cell activities. It has been shown that nobiletin facilitates cAMP/PKA/ERK/CREB signaling in hippocampal neurons, (Kawahata et al., 2013) and that it promotes glucose uptake through PKA/CREB signaling in 3T3‐F442A adipocytes (Onda, Horike, Suzuki, & Hirano, 2013). In addition, reports have demonstrated that CREB activation is associated with mitochondrial functions (Arnould et al., 2002; Herzig, Scacco, & Scarpulla, 2000; Rim & Kozak, 2002) and that nobiletin regulates mitochondrial function in neuronal cells (Jojua, Sharikadze, Zhuravliova, Zaalishvili, & Mikeladze, 2015; Wu et al., 2013). However, whether nobiletin regulates mitochondrial function in cancer cells is largely unknown.

Oral squamous cell carcinoma (OSCC) is one of the head and neck squamous cell cancers that now ranks as the sixth most prevalent cancer worldwide (Argiris, Karamouzis, Raben, & Ferris, 2008; Leemans, Braakhuis, & Brakenhoff, 2011). The 5‐year survival of OSCC patients is only about 50% although current treatments are effective against early diagnosed OSCC (Montal et al., 2016; Torre et al., 2015). Recent findings have revealed that mitochondrial function is critical for the effectiveness of chemotherapeutic agents for OSCC (Ansari et al., 2018; Liu, Xiong, Tan, & Liu, 2016). In addition, the cAMP/PKA/CREB signaling has been involved in OSCC drug resistance and migration (Chien et al., 2012; Suzuki et al., 2009).

## 2 MATERIALS AND METHODS

### 2.1 Materials

TCA‐8113 and CAL‐27 cells were obtained from the West China College of Stomatology of Sichuan University (Sichuan, China). RPMI 1640 medium, nobiletin, and Sp‐cAMP were purchased from Sigma. Fetal bovine serum was purchased from GIBCO. Cell Counting Kit‐8 (CCK8) was purchased from MCE (HY‐K0301). Anti‐PCNA (ab29), anti‐cyclin D1 (ab134175), anti‐GAPDH (ab8245), anti‐PKA (ab75993), anti‐phosphorylated‐PKA (ab32390), anti‐CREB antibody (ab32515), and anti‐phosphorylated‐CREB (ab32096) antibodies, Propidium Iodide Flow Cytometry Kit (ab139418), Glucose Uptake Assay Kit (ab136955), L‐Lactate Assay Kit (ab65331), Pyruvate Assay Kit (ab65342), and pyruvate dehydrogenase (PDH) Enzyme Activity Microplate Assay Kit (ab109902) were purchased from Abcam. Cytochrome Oxidase Activity Colorimetric Assay Kit was purchased from BioVision (K287). MitoTracker® Green FM KIT was purchased from Cell Signaling Technology (9074S).

### 2.2 Cell culture

Cells were maintained in RPMI‐1640 medium with 10% fetal bovine serum at 37°C under 95% air and 5% CO_2_.

### 2.3 Cell viability analysis

TCA‐8113 and CAL‐27 cells were treated with 50 μmol/L, 100 μmol/L, or 150 μmol/L nobiletin (purity > 98%) for 24, 48, and 72 hr, respectively. Cell numbers were determined by the CCK8 assay according to the manufacturer's instructions. The absorbance at 450 nm was detected using a microplate reader (Thermo Scientific).

### 2.4 Protein extraction and Western blot

Protein extraction and Western blot experiments were performed as previously described (Chen et al., 2016). 60 μl RIPA lysis buffer (Solarbio) was used per well of a 6‐well plate. At least three independent experiments were performed. Cell lysates were centrifuged at 18630 *g* for 15 min. The supernatants were collected and mixed with 2X SDS‐loading buffer and subjected to Western blot analysis. Protein samples were analyzed by SDS‐PAGE. Western blot was carried out using standard procedures, and immune‐reactive proteins were visualized by SuperSignal™ chemiluminescence (Thermo Scientific).

### 2.5 In vivo analysis of tumor growth

For tumor growth assays, TCA‐8113 cells were injected into the left flank of 8‐week‐old nude mice. Twenty‐four hours after implantation, 40 mg/kg nobiletin, 40 mg/kg nobiletin with 250 μg/kg Sp‐cAMP, or an equal volume of PBS were injected every 2 days for 12 days. Once palpable, tumors were measured every 2 days and volumes were calculated using the formula: a*b^2^/2 (a is the largest dimension and b is the smallest). After the mice were sacrificed, the xenografts were pictured and weighed.

### 2.6 Immunohistochemical analysis

Formalin‐fixed, paraffin‐embedded xenografts were sectioned and used for the immunohistochemical analysis. Paraffin was removed from the tissues, and the sections were hydrated through a graded series of ethanol. Antigen retrieval was performed, and sections were blocked with 5% sheep serum for 60 min. Sections were incubated with anti‐PCNA antibody overnight at 4°C. Then, signals were visualized using 3, 3’‐diaminobenzidine on the second day.

### 2.7 Flow cytometry analysis of cell cycle

Flow cytometry analysis of the cell cycle was performed by using the Propidium Iodide Flow Cytometry Kit according to the manufacturer's instructions. Briefly, cells were harvested and fixed in ethanol. Rehydrated cells were stained with propidium iodide and treated with RNase for 30 min. In the end, the cells were analyzed with MoFlo Astrios (Beckman‐Coulter).

### 2.8 Cytochrome c oxidase activity measurement

Cytochrome oxidase activities were measured with Cytochrome Oxidase Activity Colorimetric Assay Kit according to the manufacturer's instructions. The working compounds were mixed thoroughly and subjected to a measurement at 550 nm using a microplate reader (Infinite F200, Tecan). All results were normalized to the protein concentrations of the respective samples.

### 2.9 Immunofluorescent staining of mitochondria

Mitochondria were stained with MitoTracker® Green FM KIT according to the manufacturer's instructions. Images were captured with a laser‐scanning confocal microscope (True Confocal Scanner SP5; Leica; HCX Plan Apochromat confocal scanning 20×/0.7 NA objective lens) by LAS AF software (Leica).

### 2.10 Metabolism related tests

Glucose consumption experiments were performed with Glucose Uptake Assay Kit as previously described (Hai, Shin, Bi, Ye, & Jin, 2018; Sun & Zhang, 2017). Briefly, TCA‐8113 cells cultured in serum‐free medium containing 100 μmol/L nobiletin with or without 50 μmol/L Sp‐cAMP were incubated overnight in 96‐well plate with a density of 5,000 cells/well. On the next day, cells were washed and incubated in KRPH with 2% BSA for 40 min and stimulated with insulin for 20 min, followed by 2‐DG treatment for 20 min. Cells were then lysed and heat at 85°C to degrade endogenous NAD(P), followed by NADPH generation at 37°C for 1 hr. After recycling amplification reaction, the glucose uptake was quantified by optical density (OD) at 412 nm in a kinetic mode.

Lactate production was examined with L‐Lactate Assay Kit according to the manufacturer's instructions. Briefly, 1 × 10^6^ TCA‐8113 cells treated with 100 μmol/L nobiletin with or without 50 μmol/L Sp‐cAMP were harvested and washed with PBS. Then, the cells were homogenized in lactate assay buffer; after deproteinization, the samples were further incubated with reaction mix for 30 min at room temperature. Lactate production was quantified by measuring OD 450 nm.

Pyruvate production was detected with Pyruvate Assay Kit according to the manufacturer's instructions. Briefly, 1 × 10^6^ TCA‐8113 cells treated with 100 μmol/L nobiletin with or without 50 μmol/L Sp‐cAMP were harvested and homogenized in pyruvate assay buffer. After deproteinization, the samples were further incubated with the reaction mix for 30 min at room temperature. Pyruvate production was quantified by measuring OD 570 nm.

Pyruvate dehydrogenase activity was assayed using the PDH Enzyme Activity Microplate Assay Kit according to the manufacturer's instructions. Briefly, TCA‐8113 cells treated with 100 μmol/L nobiletin with or without 50 μmol/L Sp‐cAMP were harvested and total proteins were extracted. Then, 200 µl of 13.5 mg/ml proteins was loaded onto measuring plates and incubated for 3 hr at room temperature. After rinsing with the stabilizer solution, samples were incubated with the assay solution and the kinetics of OD at 450 nm was read for 30 min.

### 2.11 Statistical analysis

All experiments were repeated at least three times, and all data are presented as means ± *SD*. Statistical analyses were performed with GraphPad Prism 5.0 (GraphPad Software, Inc.). The statistical comparisons were performed by one‐way ANOVA followed by Tukey's post hoc test. *p* < .05 was considered to indicate statistically significant differences. *p < .05, **p < .01.

## 3 RESULTS

### 3.1 Nobiletin treatment inhibits OSCC cell proliferation

**Figure 1:**
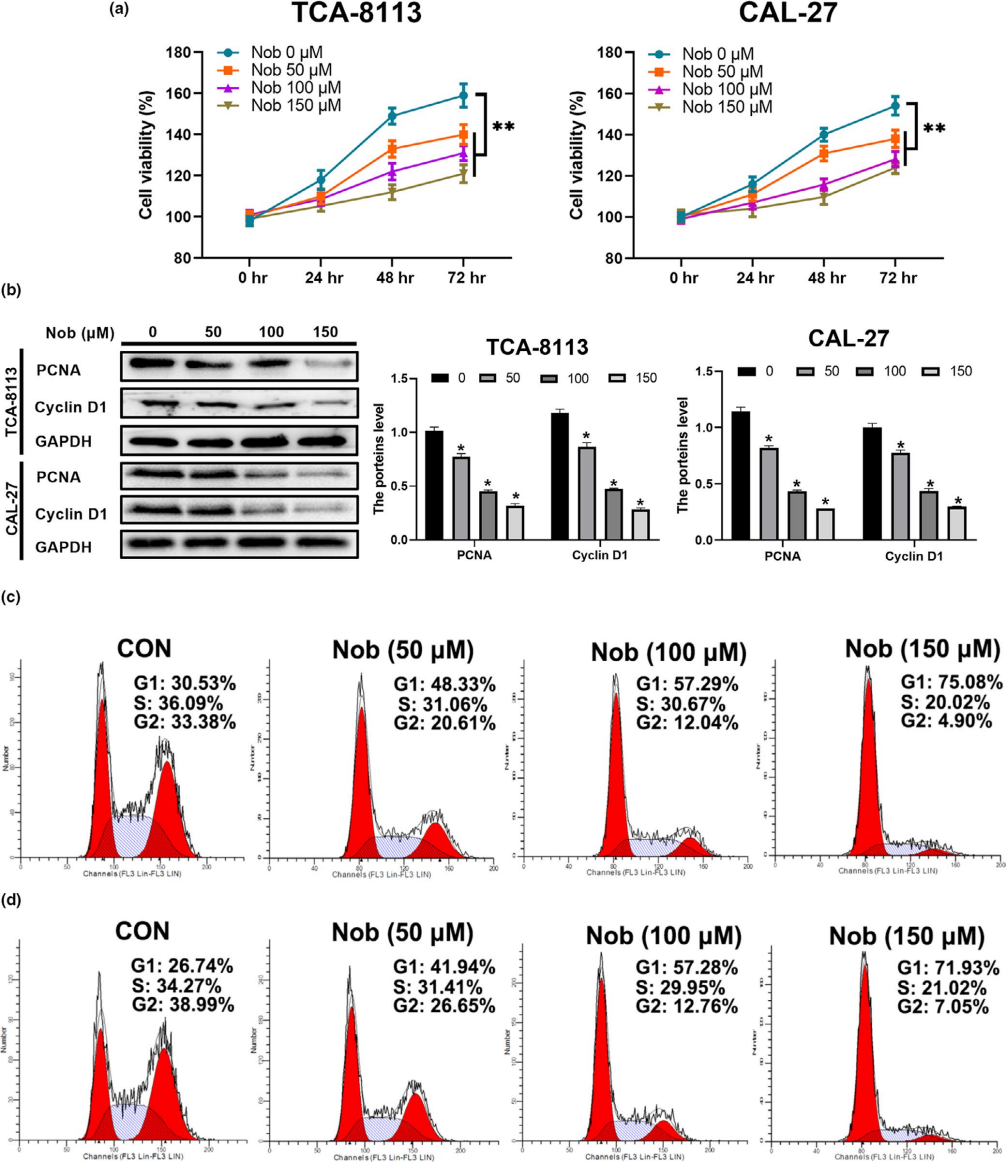
Nobiletin treatment inhibits OSCC cell proliferation. (a) TCA‐8113 and CAL‐27 cells were treated with the indicated concentrations of nobiletin for 24, 48, and 72 hr. Cell numbers were determined by Cell Counting Kit‐8 (CCK8). Data from 3 independent experiments are shown as mean ± *SD*. ***p < .001 compared to control group at the same time point. (b) Proliferation‐related protein levels were examined by Western blot. GAPDH was used as loading control. Representative images were taken from three independent experiments. (c, d) Cell cycle analysis of TCA‐8113 (c) and CAL‐27 cells (d) treated with the indicated concentrations of nobiletin for 24 hr was performed by flow cytometry. Cells treated with an equal volume of DMSO served as control

To study the effect of nobiletin on OSCCs, we treated the OSCCs cell lines TCA‐8113 and CAL‐27 with 50, 100, or 150 μmol/L of nobiletin. We first analyzed the viability of TCA‐8113 and CAL‐27 cells treated with nobiletin or control DMSO. The results showed that nobiletin significantly inhibited cell viability at all concentrations (Figure 1a). However, we did not observe any significant changes in cell viability when OSCC cells were treated with nobiletin at concentrations lower than 50 μmol/L (data not shown). Additionally, we examined the expression levels of proliferation‐related proteins PCNA and cyclin D1 and found that with the increase in nobiletin concentration, their protein levels were gradually declined in both TCA‐8113 and CAL‐27 cells (Figure 1b). Moreover, we analyzed the cell cycle status of cells treated with nobiletin or DMSO. Interestingly, the flow cytometry results showed that nobiletin treatment dramatically attenuated the percentage of cells in the S and G2 phases (Figure 1c). In addition, higher nobiletin concentrations resulted in fewer cells in the G2 and S phases and in a significant enrichment of cells in the G1 phase (Figure 1c). Moreover, similar results were obtained following treatment of CAL‐27 cells with nobiletin. Taken together, our results indicated that nobiletin inhibited cell proliferation via arresting cell cycle at the G1 phase.

### 3.2 Nobiletin treatment inhibits PKA/CREB pathway

**Figure 2:**
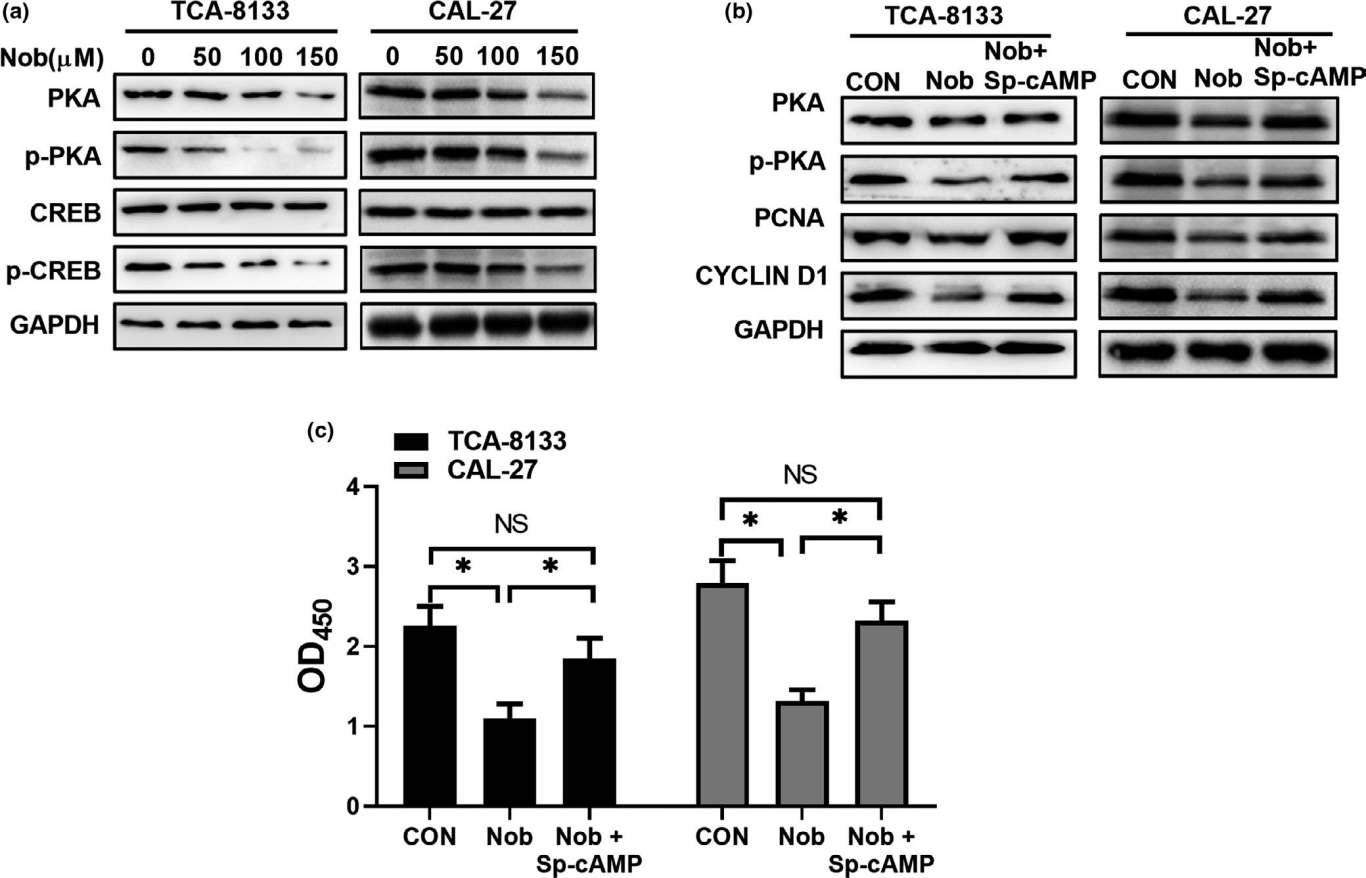
Nobiletin treatment inhibits PKA/CREB pathway. (a) Western bolt analysis of key PKA pathway markers in TCA‐8133 and CAL‐27 cells following treatment with the indicated concentrations of nobiletin for 24 hr. Cells treated with an equal volume of DMSO served as control. (b) TCA‐8113 and CAL‐27 cells were treated with 100 μmol/L nobiletin for 24 hr in the absence or presence of 50 μmol/L Sp‐cAMP, a potent PKA activator, followed by Western blot analysis of proliferation‐related proteins. Cells treated with an equal volume of DMSO served as control. Representative images were taken from three independent experiments. (c) Proliferation of TCA‐8133 and CAL‐27 cells was determined by the Cell Counting Kit‐8 (CCK8) after 24 hr treatment with 100 μmol/L nobiletin in the absence or the presence of 50 μmol/L Sp‐cAMP. Cells treated with equal volume of DMSO served as control. Data from three independent experiments shown as mean ± *SD*. *, *p* < .05 compared to control group

To further dissect the molecular mechanism underlying nobiletin‐mediated proliferation inhibition, we analyzed the PKA/CREB pathway. Interestingly, our results showed that nobiletin treatment of TCA‐8113 and CAL‐27 cells resulted in a dose‐dependent decrease in the levels of PKA and phosphorylated‐PKA (Figure 2a). As a result, the levels of phosphorylated‐CREB were severely decreased (Figure 2a), while the total levels of CREB were not affected, suggesting that nobiletin‐mediated inhibition of PKA activity further interfered with the phosphorylation of CREB. Importantly, 50 μmol/L Sp‐cAMP, a potent PKA activator, restored the expression of PKA, phosphorylated‐PKA, PCNA, and cyclin D1 (Figure 2b). Besides, our results showed that Sp‐cAMP restored the viability of nobiletin‐treated cells (Figure 2c). These results further suggested that nobiletin functions through inhibiting PKA signaling in OSCC.

### 3.3 Nobiletin treatment inhibits mitochondrial activity

**Figure 3:**
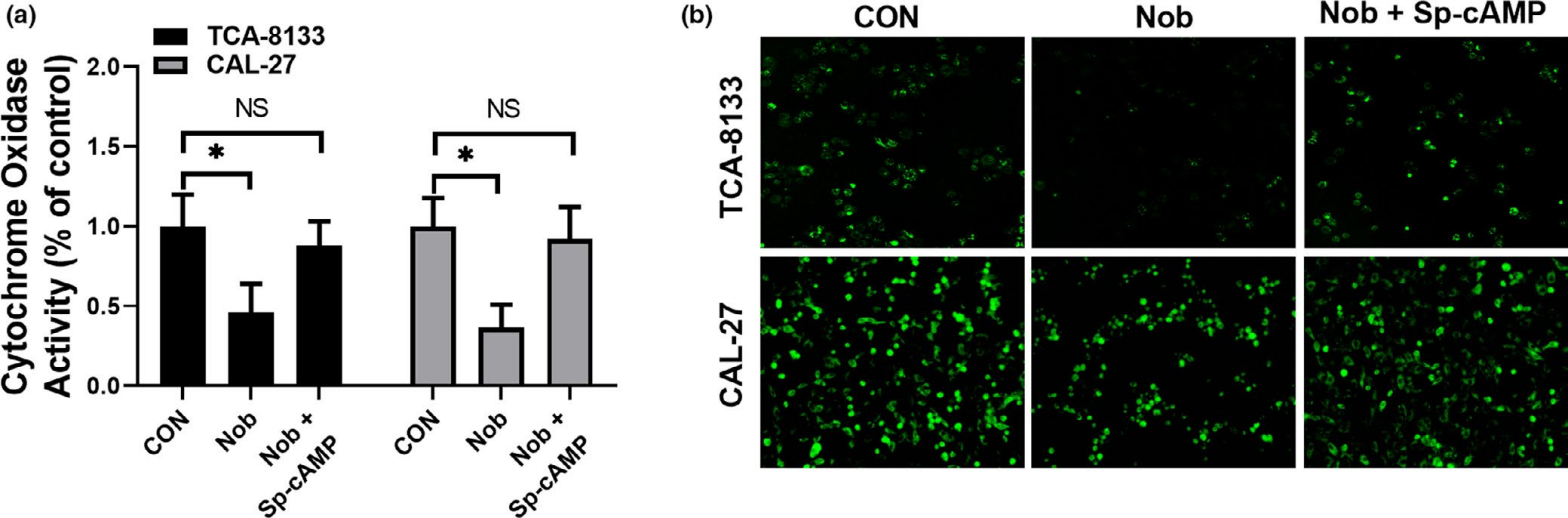
Nobiletin treatment inhibits mitochondrial activity. (a) TCA‐8113 and CAL‐27 cells were incubated with 100 μmol/L nobiletin for 24 hr in the absence or the presence of 50 μmol/L Sp‐cAMP, and then cytochrome oxidase activity was measured. Data from three independent experiments are shown as mean ± *SD*. *p < .05 compared to control group. (b) Representative immunofluorescent images of TCA‐8113 and Cal‐27 cells labeled with MitoTracker Green. Images were captured at 200× magnification

Since PKA/CREB activity is directly associated with mitochondrial activity (Xie et al., 2018), we next evaluated the mitochondrial activity of OSCC cells treated with nobiletin. Our results showed that the cytochrome oxidase activity was significantly impaired when TCA‐8113 and CAL‐27 cells were treated with nobiletin, and administration of Sp‐cAMP significantly restored cytochrome oxidase activity (Figure 3a). Moreover, MitoTracker Green staining showed that nobiletin treatment resulted in less alive and metabolically active mitochondria and administration of Sp‐cAMP rescued the number of functional mitochondria (Figure 3b) suggesting that nobiletin reduced the mitochondrial number and inhibited their activity in OSCC cells.

### 3.4 Nobiletin treatment inhibits aerobic glycolysis in OSCC cells

**Figure 4:**
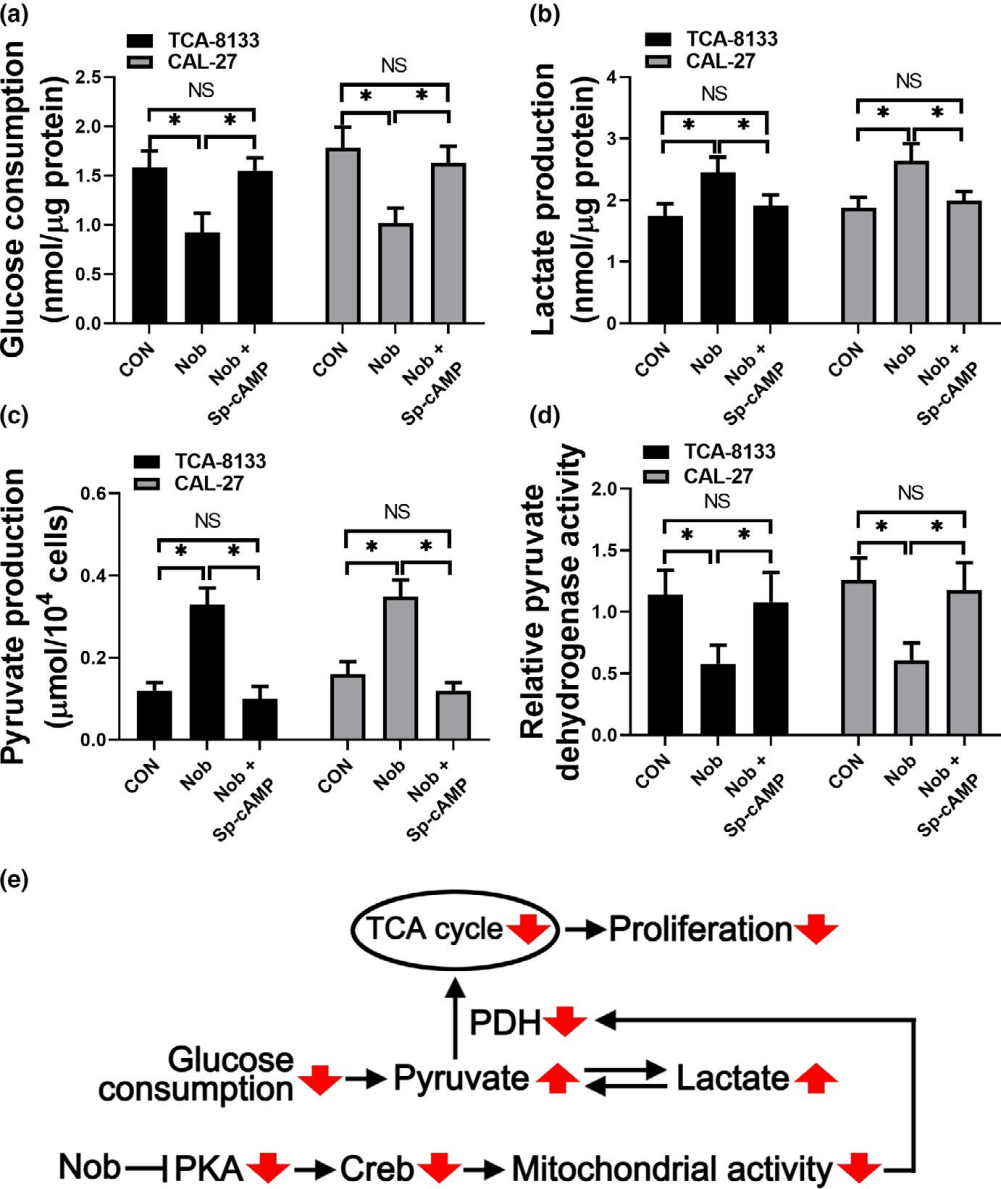
Nobiletin treatment inhibits aerobic glycolysis in TCA‐8113 and CAL‐27 cells. TCA‐8113 and CAL‐27 cells were incubated with 100 μmol/L nobiletin for 24 hr in the absence or the presence of 50 μmol/L Sp‐cAMP. Glucose consumption (a), lactate production (b), and pyruvate production (c) in the cell culture medium were analyzed. Data from three independent experiments are shown as mean ± *SD*. *p < .05 compared to control group. (d) Total pyruvate dehydrogenase activity was assessed in TCA‐8113 and CAL‐27 cells. Data from three independent experiments were normalized to control group and are shown as mean ± *SD*. *p < .05 compared to control group. (e) A possible model indicating that nobiletin inhibits PKA/CREB signaling and thus contributes to the impeded glycolysis in OSCC cells

Apart from the mitochondrial activity, glucose consumption is another index used to evaluate the respiration process. Thus, we next analyzed the metabolic alterations in TCA‐8113 and CAL‐27 cells treated with 100 μmol/L nobiletin with or without 50 μmol/L Sp‐cAMP. Interestingly, our results showed that when OSCC cells were treated with nobiletin, glucose consumption and pyruvate dehydrogenase activity were significantly reduced, while lactate and pyruvate production were significantly increased (Figure 4a–d). Importantly, treatment with Sp‐cAMP attenuated the metabolic effects of nobiletin, which returned to the basal levels (Figure 4a–d), suggesting that nobiletin‐mediated metabolic changes mainly relied on the cAMP pathway. Taken together, our results suggested a model where nobiletin inhibited the PKA/CREB pathway and in turn impeded mitochondrial activity. As a result, impaired pyruvate dehydrogenase activity increased the accumulation of pyruvate, which inhibited the tricarboxylic acid cycle (TCA) cycle and finally inhibited proliferation (Figure 4e).

### 3.5 Nobiletin treatment inhibits OSCC tumor formation in vivo

**Figure 5:**
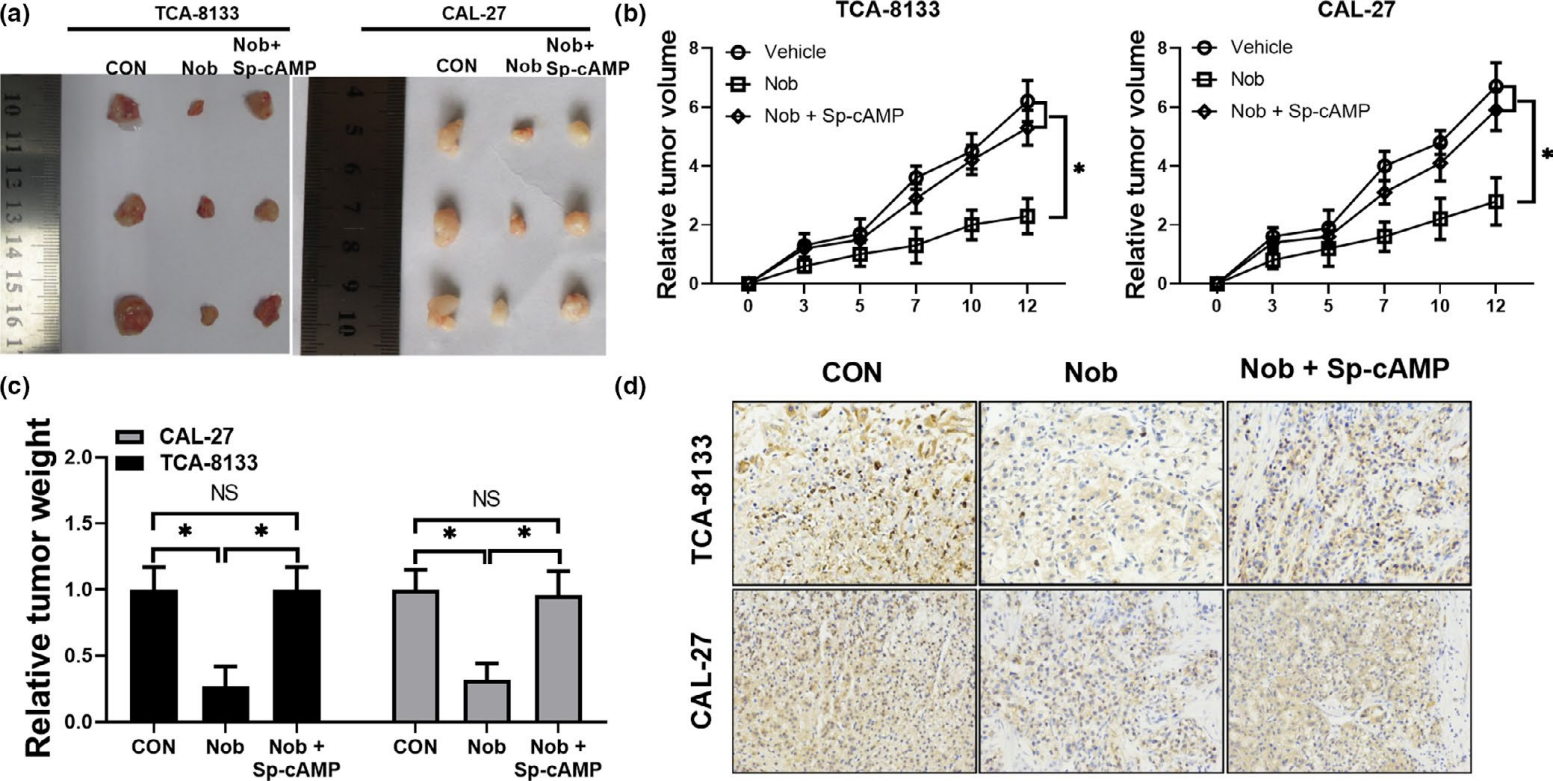
Nobiletin treatment inhibits tumor growth in OSCC models via inhibiting the PKA pathway. TCA‐8133 and CAL‐27 cells were injected subcutaneously into the left flank of nude mice. 24 hr after implantation, 40 mg/kg nobiletin, 40 mg/kg nobiletin with 250 μg/kg Sp‐cAMP, or an equal volume of PBS were injected every 2 days for 12 days. (a) Representative tumors from nude mice treated with nobiletin, nobiletin with Sp‐cAMP, or PBS. Average tumor volumes (b) and weights (c) of TCA‐8133 and CAL‐27 xenografts. (d) Representative images of immunohistochemical analysis of PCNA expression in TCA‐8133 and CAL‐27 xenografts (200×)

Next, we explored the anti‐proliferative function of nobiletin in vivo. TCA‐8133 and CAL‐27 cells were injected subcutaneously into the left flank of nude mice. Twenty‐four hours after implantation, 40 mg/kg nobiletin, 40 mg/kg nobiletin with 250 μg/kg Sp‐cAMP, or an equal volume of PBS were injected into the mice every 2 days for 12 days. The xenografts generated in mice injected with nobiletin were significantly reduced in both volume and weight compared to the xenografts generated in control mice (Figure 5a–c). Interestingly, Sp‐cAMP injection reversed nobiletin‐mediated proliferation inhibition (Figure 5a–c), suggesting that high cAMP levels in the tumors of patients may abate the function of nobiletin. In addition, we analyzed PCNA expression levels by immunohistochemical staining and our results revealed that nobiletin treatment resulted in a dramatic decrease in PCNA levels, suggesting that proliferation was significantly inhibited by nobiletin (Figure 5d). As expected, Sp‐cAMP reversed the anti‐proliferative effect of nobiletin as revealed by PCNA staining (Figure 5d).

Taken together, our results demonstrated that nobiletin inhibited OSCC proliferation via regulating PKA/CREB pathway both in vitro and in vivo. Mechanistically, reduced levels of phosphorylated‐CREB led to a decrease in mitochondrial activity and glucose consumption. However, nobiletin administration resulted in pyruvate and lactate accumulation. These metabolic alterations resulted in a significant decrease in OSCC cell proliferation.

## 4 DISCUSSION

Incidence of oral cancer accounts for approximately 2.1% of all cancers (Nishiyama et al., 2018). The discovery of chemicals with better clinical efficacy is an ultimate goal for drug development (Wu, Song, et al., 2018). Nobiletin is a common flavone enriched in daily consumed fruits such as oranges. Here, our data showed that nobiletin alone was able to restrain the growth of OSCC cells through inducing cell cycle arrest. When OSCC cells were treated with 150 μmol/L nobiletin, the G2 cell population decreased from 33.38% to 4.90% and the expression of PCNA and cyclin D1 was almost hardly detectable. Our data agree with previous data that nobiletin inhibits cell growth via inducing G1 cell cycle arrest in a dose‐dependent manner (Lee et al., 2017; Lien et al., 2016; Morley et al., 2007; Uesato et al., 2014). Our data further validated the anti‐proliferative role of nobiletin and suggested that nobiletin alone can be utilized as an anti‐cancer drug. However, both previous reports and our data did not explore the cytotoxicity of nobiletin on normal cells at the concentration of 150 μmol/L, although the in vivo assay did not reveal any abnormalities in the mice except that nobiletin restrained the growth of xenografts (Figure 5). Future studies may examine nobiletin toxicity in normal cells.

At the molecular level, our data illustrated that nobiletin reduced the protein levels of both PKA and phosphorylated‐PKA. However, treatment of PC12 cells with 50 µmol/L nobiletin (Lai et al., 2011) and hippocampal neurons with 100 µmol/L nobiletin for less than 15 min augmented PKA signaling (Matsuzaki et al., 2008). In this study, nobiletin concentrations less than 50 μmol/L did not have a significant effect (data not shown). Further, concentrations of nobiletin higher than 50 μmol/L resulted in significant inhibition of OSCC cell proliferation in vitro. Besides, we demonstrated, in 2 OSCC cell lines, that nobiletin decreased the protein levels of PKA. The differences may also be due to different cell types. Here, we found that oral administration of 40 mg/kg nobiletin inhibited in vivo OSCC tumor formation, which is consistent with previous studies that low‐dose of nobiletin (20 mg and 40 mg/kg oral administration) is effective in controlling tumor progression in different disease models (Da et al., 2016; Xie et al., 2019). Nonetheless, our data demonstrated through various experiments that Sp‐cAMP, a PKA activator, functions against nobiletin, which validated our results that the PKA/CREB pathway is impaired when OSCC cells were treated with nobiletin. More importantly, our data revealed that mitochondrial function was dramatically affected by nobiletin. It has been reported that the PKA/CREB pathway tightly regulates mitochondrial functions (J. Lee et al., 2005; Rim & Kozak, 2002). Thus, our data link nobiletin, CREB, and mitochondrial function in OSCC cells, which may illuminate a novel aspect of OSCC treatment. However, since nobiletin severely impaired mitochondrial function, it is worth exploring how much influence nobiletin has on mitochondria in normal cells. Hence, our results showed that the respiration process of OSCC cells was modulated by the administration of nobiletin, which is consistent with previous reports suggesting that CREB positively regulates mitochondrial activity and glucose consumption.

Metabolically, nobiletin suppressed glucose consumption and induced lactate and pyruvate production via inhibiting pyruvate dehydrogenase activity. These results strongly suggested that nobiletin inhibited aerobic glycolysis in TCA‐8113 cells. Most importantly, our data provided in vivo data in a mouse model where nobiletin inhibited the growth of xenografts. The size and weight of xenografts as well as the number of PNCA positive cells inside the xenografts were significantly reduced suggesting a promising clinical prospect of nobiletin.

In summary, our data validated the antitumor function of nobiletin in OSCC cells through inhibiting proliferation. Additionally, our data demonstrated that nobiletin functions via the PKA/CREB pathway and modulates mitochondrial function. Most importantly, nobiletin inhibited xenografts' growth in vivo. These data suggested that nobiletin is a promising drug candidate for OSCC treatment.

### 4.1 Statement of human and animal rights

The experimental procedures in this study were conducted in accordance with the Ethics Committee of the Wenzhou Medical University's approved protocols (wydw2017‐0126).

## CONFLICT OF INTEREST

The authors declare no conflict of interest.
